# Internet of medical things-enabled CRISPR diagnostics for rapid detection of SARS-CoV-2 variants of concern

**DOI:** 10.3389/fmicb.2022.1070940

**Published:** 2022-11-18

**Authors:** Huihuang Lin, Weibo Zheng, Shenwei Li, Yu Wang, Dong Wei, Leiying Xie, Wei Lu, Zhengan Tian, Shaowei Wang, Jieming Qu, Jia Liu

**Affiliations:** ^1^Department of Pulmonary and Critical Care Medicine, Ruijin Hospital, Shanghai Jiao Tong University School of Medicine, Shanghai, China; ^2^Institute of Respiratory Diseases, Shanghai Jiao Tong University School of Medicine, Shanghai, China; ^3^Shanghai Key Laboratory of Emergency Prevention, Diagnosis and Treatment of Respiratory Infectious Diseases, Shanghai, China; ^4^State Key Laboratory of Infrared Physics, Shanghai Institute of Technical Physics, Chinese Academy of Sciences, Shanghai, China; ^5^University of Chinese Academy of Sciences, Beijing, China; ^6^Shanghai International Travel Healthcare Center, Shanghai, China; ^7^Shanghai Institute for Advanced Immunochemical Studies and School of Life Science and Technology, ShanghaiTech University, Shanghai, China; ^8^Department of Infectious Diseases, Research Laboratory of Clinical Virology, Ruijin Hospital, Shanghai Jiao Tong University School of Medicine, Shanghai, China; ^9^School of Physical Science and Technology, ShanghaiTech University, Shanghai, China; ^10^Shanghai Clinical Research and Trial Center, Shanghai, China; ^11^Gene Editing Center, School of Life Science and Technology, ShanghaiTech University, Shanghai, China; ^12^Guangzhou Laboratory, Guangzhou International Bio Island, Guangzhou, Guangdong, China

**Keywords:** SARS-CoV-2, variants of concern, CRISPR-Cas12a, closed-lid, smartphone control, point-of-care testing, internet of medical things

## Abstract

Previous studies have highlighted CRISPR-based nucleic acid detection as rapid and sensitive diagnostic methods for severe acute respiratory syndrome coronavirus 2 (SARS-CoV-2). Here, we reported an optimized CRISPR-Cas12a diagnostic platform for the safe and rapid detection of SARS-CoV-2 variants of concern (VOCs). This platform, which was referred to as CALIBURN-v2, could complete the diagnosis on extracted RNA samples within 25 min in a closed-lid reaction mode and had 100-fold increase in detection sensitivity in comparison with previous platforms. Most importantly, by integrating a portable device and smartphone user interface, CALIBURN-v2 allowed for cloud server-based data collection and management, thus transforming the point-of-care testing (POCT) platform to internet of medical things (IoMT) applications. It was found that IoMT-enabled CALIBURN-v2 could achieve 95.56% (172 out of 180) sensitivity for SARS-CoV-2 wild type and 94.38% (84 out of 89) overall sensitivity for SARS-CoV-2 variants including Delta and Omicron strains. Therefore, our study provides a feasible approach for IoMT-enabled CRISPR diagnostics for the detection of SARS-CoV-2 VOCs.

## Introduction

The outbreak of coronavirus disease 2019 (COVID-19) (Petersen et al., 2020) has become a major challenge to the public health worldwide. The pathogen causing COVID-19 is severe acute respiratory syndrome coronavirus 2 (SARS-CoV-2; Wu, F. et al., 2020; Zhou et al., 2020). As of 15 October 2022, there were more than 620 million confirmed cases and 6.5 million deaths according to World Health Organization (WHO, 2022a). One challenge in controlling COVID-19 pandemic is to have accurate diagnosis of SARS-CoV-2, which can be easily confused with other respiratory pathogens such as influenza virus (Wang et al., 2020). Currently, reverse transcription quantitative polymerase chain reaction (RT-qPCR) is the gold standard for the nucleic acid diagnosis of COVID-19 (Wu, J. et al., 2020). However, RT-qPCR requires specialized instruments, thus restricting its applications to centralized facilities.

To overcome the above problem, isothermal amplification-based nucleic acid detection methods have been developed for SARS-CoV-2 detection, including loop-mediated isothermal amplification (LAMP; Notomi et al., 2000) and recombinase polymerase amplification (RPA; Piepenburg et al., 2006). However, these methods are known to be associated with nonspecific amplification products (Obande and Banga Singh, 2020).

Recent studies have highlighted clustered regularly interspaced short palindromic repeats (CRISPR)/CRISPR-associated (Cas) systems as point-of-care testing (POCT) platforms for rapid detection of pathogens (Abudayyeh et al., 2016; Chen et al., 2018; Li et al., 2018). CRISPR-based nucleic acid detection relies on the collateral cleavage activity of Cas12a, Cas13a or equivalent nucleases. Importantly, the activation of the collateral activity of Cas nucleases was dependent on the complementary base pairing between CRISPR RNA (crRNA) and the target nucleic acid sequences, which ensures the specificity and sensitivity of the detection assay. CRISPR-based assays such as SHERLOCK (Specific High-sensitivity Enzymatic Reporter UnLOCKing; Kellner et al., 2019) or DETECTR (DNA Endonuclease-Targeted CRISPR Trans Reporter; Chen et al., 2018) have been adopted to detect SARS-CoV-2 (Broughton et al., 2020; Joung et al., 2020). These methods can be completed within 30–60 min without the need of specialized instruments and are thus feasible choice for POCT application.

In a previous study, we have developed a CRISPR-based platform, named as CALIBURN for Cas12a-Linked Beam Unlocking Reaction, for the rapid and sensitive detection of respiratory pathogens. In the current study, we developed an extensively optimized Cas12a reaction based on CALIBURN and named it CALIBURN-v2 following our previous work. In CALIBURN-v2, repeated capping and uncapping steps were avoided, which could reduce the risk of aerosol contaminant (Wang et al., 2019; Xiong et al., 2020) during the handling procedures. Moreover, we developed a portable fluorescence detection device and companion smartphone application for CALIBURN-v2. These new features allowed the data collected from CALIBURN-v2 detection to be uploaded to cloud servers for subsequent data analysis and management (Guo et al., 2021), thus enabling an internet of medical things (IoMT) approach for SARS-CoV-2 diagnostics.

## Materials and methods

### Clinical samples

SARS-CoV-2 samples were collected by Shanghai International Travel Healthcare Center and Ruijin Hospital. Influenza A virus (IAV), Influenza B virus (IBV) and negative samples were collected by Ruijin Hospital.

### RNA extraction

Nasopharyngeal (NP) swab was stored in viral transport medium (VTM) (Yocon Biology, Beijing, China) and RNA was extracted using TIANamp Virus DNA/RNA Kit (TIANGEN, Beijing, China) according to the manufacturer’s instructions.

### Reverse transcription quantitative PCR (RT-qPCR) experiments

RT-qPCR was performed using One Step PrimeScript RT-PCR Kit (Takara, China) in QuantStudio 6 Flex System thermocycler (Applied Biosystems, United States). Primers were designed according to the instructions of Chinese Centers for Disease Control (CDC; Supplementary Table S1). The cycling conditions of the reactions were as follows: reverse transcription reaction at 42°C for 5 min, heat activation at 95°C for 10 s and 40 cycles of denaturation at 95°C for 5 s followed by annealing and elongation at 60°C for 34 s.

### Reverse transcription recombinase polymerase amplification (RT-RPA) reactions

Primers of SARS-CoV-2 N gene were designed and screened for optimum efficiency in RT-RPA reactions (Supplementary Table S1). Briefly, combination of different forward and reverse primer pairs were used to amplify SARS-CoV-2 N gene in RT-RPA reactions and the product was subjected to Cas12a fluorescence reaction. The readout of Cas12a reaction was accounted for evaluating the yield of RT-RPA reactions. The optimum RT-RPA primer pairs were used for subsequent RT-RPA reactions.

### Preparation of crRNA and RNA target

For crRNA preparation, *in vitro* transcription (IVT) template was generated by annealing a T7-3G primer (Supplementary Table S1) to an antisense oligonucleotide containing an antisense T7 promoter, a crRNA repeat and a spacer sequence. For RNA target production, the target sequences of IAV-M, IBV-HA and SARS-CoV-2-N genes were cloned into pUC57 plasmid and then PCR amplified using primers containing a T7 promoter. PCR products were confirmed by gel electrophoresis and used as the IVT template after purification *via* Gel Extraction Kit (Omega, United States). crRNA and target RNA were transcribed using the above IVT templates and HiScribe T7 Quick High Yield RNA Synthesis Kit (NEB, USA) at 37°C overnight. The DNA templates in the IVT reactions were removed by treatment with deoxyribonuclease I (DNase I), and the RNA product was purified by phenol: chloroform extraction followed by ethanol precipitation. The concentration of purified RNA was quantified by Nanodrop spectrophotometer, and the copy number was calculated using the following formula: RNA copy number (copies/μL) = [6.02 × 10^23^ (copies/mol) × RNA concentration (g/μL)]/molecular weight of full transcript (g/mol).

### Coupled RT-RPA and Cas12a reaction

The coupled RT-RPA and Cas12a reaction was optimized based on a previous study (Wang et al., 2021). RT-RPA reaction was performed with a commercial kit (AmpFuture, China) according to the manufacturer’s protocol. Briefly, a 25 μl reaction containing 14.7 μl rehydration buffer, 5 μl RNA samples, 0.4 μM of each primer and 14 mM magnesium acetate was incubated at 42°C for 30 min. The following Cas12a-based fluorescence activation was performed in a 20 μl reaction containing 5 μl of RT-RPA products, 2 μl of 10 × NEBuffer 3.1, 100 nM crRNA, 50 nM Cas12a and 25 μM single-stranded DNA (ssDNA) reporter probe and incubated at 37°C for 30 min. The fluorescence signal was monitored using SpectraMax iD3 Multi-Mode Microplate Reader with an excitation wavelength of 485 nm and an emission wavelength of 520 nm. The Cas12a protein used in this study is derived from *Lachnospiraceae bacterium* ND2006 (LbCas12a; Chen et al., 2018).

To maximize the efficiency of coupled RT-RPA and Cas12a reaction, we optimized several reaction parameters as follows. For Cas12a reaction, the reaction time ranging from 5 to 20 min and the temperature ranging from 35 to 45°C were evaluated. For the buffering conditions in Cas12a reaction, we optimized the concentrations of Na^+^ ranging from 0 to 100 mM, Mg^2+^ ranging from 0 to 25 mM, Tris–HCl ranging from 0 to 100 mM, dithiothreitol (DTT) ranging from 0 to 10 mM and bovine serum albumin (BSA) ranging from 0 to 500 μg/ml. For RT-RPA optimization, ribonuclease H (RNase H) concentrations ranging from 0 to 1.0 U/μL were evaluated. For RT-RPA and Cas12a reaction coupling, we optimized the ratio of reaction volume between RT-RPA and Cas12a ranging from 1:1 to 1:5. In addition, we optimized the sequences of ssDNA reporters containing five consecutive A, T, C or G, respectively (Supplementary Table S1).

For all the above optimization experiments, the following conditions were kept consistent including the RT-RPA kit used, 100 nM crRNA, 50 nM LbCas12a and 1.25 μM ssDNA reporter probes. The optimization process was carried out in a consecutive or cumulative manner, with a single parameter being optimized in each round of experiment and the determined optimal conditions being applied to the next round of experiment. The criteria for optimum parameters are improvement of fluorescence signal readout from Cas12a reaction or lower limit-of-detection (LOD) values.

### CALIBURN-v2 reaction

With the above optimized reaction parameters, we sought to establish a streamlined RT-RPA and Cas12a reaction (CALIBURN-v2) as follows. RT-RPA was performed in a 25 μl reaction mixture containing 14.7 μl rehydration buffer, 5 μl RNA samples, 0.4 μM of each primer, 14 mM magnesium acetate and 0.1 U/μL RNase H and reaction incubated at 42°C for 15 min. Subsequent Cas12a reaction was performed directly with the above 25 μl RT-RPA reaction product and supplemented with additional components including 10 mM Tris–HCl, pH 7.9, 20 mM NaCl, 15 mM MgCl_2_, 0.5 mM DTT, 100 μg/ml BSA, 100 nM crRNA, 50 nM LbCas12a and 1.25 μM 5C-FQ reporter probe in a total reaction volume of 100 μl. The Cas12a reaction was incubated at 42°C for 10 min.

### CALIBURN-v2 in a closed-lid mode

The closed-lid mode was achieved by syringe injection of 75 μl pre-made Cas12a reaction master mixture into the 25 μl RT-RPA reaction product following CALIBURN-v2 reaction conditions as described above in section 2.7, without re-opening the lid of the reaction tube.

### Design of IoMT-enabled point-of-care testing device and smartphone application with cloud function of data management

We developed a CALIBURN-v2-compatible instrument system containing three modules: a portable POCT device for CALIBURN-v2 reaction, a wireless communication module and a cloud platform display module. The portable POCT device for CALIBURN-v2 reaction signal readout was designed to be a compact, customized box with a size of 20 cm × 8 cm × 5 cm. This box contained integrated blue light-emitting diode (LED) as the excitation light source equipped with a light bandpass filter, and a photoelectric sensor as the fluorescence detector which was equipped with a fluorescence filter to maximize the signal-to-noise ratio. The fluorescence signal of CALIBURN-v2 reaction was collected, converted to electric signal and sent to wireless communication unit by Bluetooth. The wireless communication module consisted of wireless communication unit and position information acquisition unit, which was used to obtain the global positioning system (GPS) information and communicate with the cloud platform. The cloud platform received three kinds of information from the wireless communication module: the time of detection actions, location information and results of detection. The cloud platform could display the results on a map according to the GPS coordinates and meanwhile formed a database for statistics.

To execute the signal reading, a tube containing negative control samples was first placed into the sample chamber. The application terminus was launched on smartphone and data were acquired by clicking “Calibration” button. This process enabled determination of the fluorescence threshold for a positive sample. Test samples were then loaded into the sample chamber and fluorescence could be collected by clicking “Detection” button. The output results were displayed as “Positive” or “Negative” symbol and uploaded into a cloud server for real-time data management.

### Statistical analysis

The experimental data were analyzed using GraphPad Prism 8. Two-sided confidence intervals of sensitivity, specificity, positive predictive agreement (PPA) and negative predictive agreement (NPA) were calculated using Clopper-Pearson method.

## Results

### Screening RT-RPA primers and Cas12a probes for coronaviruses and influenza viruses

**Figure 1:**
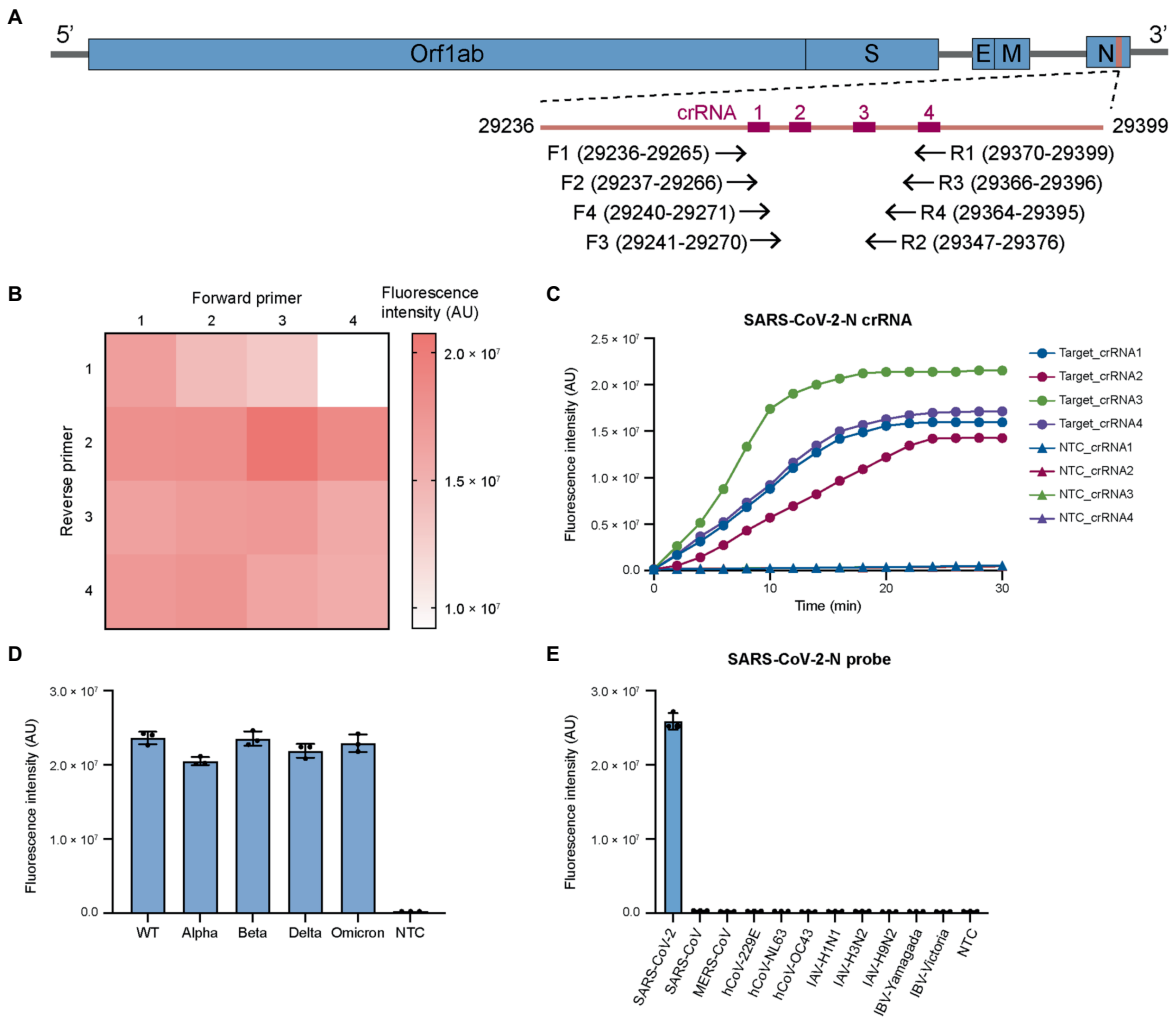
Screening RT-RPA primers and CRISPR crRNAs for coronaviruses and influenza viruses. **(A)** Genomic structure of SARS-CoV-2 showing the target sites in N gene for RT-RPA primers and Cas12a crRNAs. The primers and crRNAs are designed to target conserved region. **(B)** Screening RT-RPA primers for SARS-CoV-2 detection. **(C)** Screening crRNAs for SARS-CoV-2 detection. **(D)** The universal detection for SARS-CoV-2 variants. **(E)** The specificity of SARS-CoV-2 detection. For each virus, the viral copies are in the range of 10^6^–10^7^ per reaction. The RT-RPA primers are SARSCoV2-N-RPA-F3/SARSCoV2-N-RPA-R2 and crRNA is SARSCoV2-N-crRNA3. WT, wild-type. Target, SARS-CoV-2 IVT N gene RNA (1 nM). NTC, non-template control. The data from three biological replicates are shown as mean ± standard deviation (SD).

WHO has listed five variants of concern (VOCs) which have increased transmissibility or virulence, including B.1.1.7 (Alpha), B.1.351 (Beta), P.1 (Gamma), B.1.617.2 (Delta) and B.1.1.529 (Omicron; Walensky et al., 2021; WHO, 2022b). We analyzed the genomic sequences of existing SARS-CoV-2 strains and identified a highly specific region in N gene (29,236–29,399) for designing RT-RPA primers and Cas12a crRNAs (Figure 1A; Supplementary Figure S1A; Supplementary Table S1). This genomic sequence is conserved among all SARS-CoV-2 variants but are specific to SARS-CoV-2 with distinct features from other commonly seen respiratory pathogens. These crRNAs were also examined for binding sites in human genome and no significant similarity sequences were identified.

We then designed four forward and four reverse primers and screened the 16 primer combinations to determine optimal primer pairs for RT-RPA reactions. The efficiency of RT-RPA amplification was quantified by a coupled Cas12a fluorescence activation reaction using a fixed crRNA. Several primer pairs that could generate high level of fluorescence were determined (Figure 1B) with the combination of SARSCoV2-N-RPA-F3/SARSCoV2-N-RPA-R2 giving the highest signal. Based on the optimal RT-RPA primer pair, we designed four crRNAs for Cas12a reaction. The fluorescence *trans*-cleavage assay showed that SARSCoV2-N-crRNA-3 had the strongest collateral activity (Figure 1C). We then sought to examine the optimal RT-RPA primers and Cas12a crRNA on SARS-CoV-2 variants. The results revealed similar performance of the primers and crRNAs on wild-type and mutant SARS-CoV-2 (Figure 1D), demonstrating that the design of primers and crRNA for targeting the conserved SARS-CoV-2 region is successful.

Next, we examined RT-RPA primers and Cas12a crRNA for their specificity across different coronaviruses. Sequence alignment revealed that there were at least multiple nucleotide differences in the selected target site in the N gene between SARS-CoV-2 and other human coronaviruses, including SARS-CoV, MERS-CoV, hCoV-NL63, hCoV-229E, hCoV-OC43, and hCoV-HKU1 (Supplementary Figure S1B). Importantly, SARS-CoV and MERS-CoV, which are closely related with SARS-CoV-2 in nucleotide sequence, had several nucleotide variations with SARS-CoV-2 in protospacer adjacent motif (PAM) of designed crRNA (Supplementary Figure S1B). The difference in PAM sequence could help to reduce the cross reactivity between SARS-CoV-2 and SARS-CoV or MERS-CoV. The results showed that our design could distinguish SARS-CoV-2 and other coronaviruses with notable specificity (Figure 1E). In addition, SARS-CoV-2 primers and crRNAs had little cross reactivity with various influenza virus A (IAV) and influenza virus B (IBV) serotypes (Figure 1E). Overall, the above results showed that by carefully designing RT-RPA primers and Cas12a crRNAs, broad-spectrum detection of SARS-CoV-2 variants could be achieved without cross reactivity with other common respiratory viruses. This feature was particularly important given the constantly evolving SARS-CoV-2 (Plante et al., 2021).

### Design of CALIBURN-v2 for one-pot, closed-lid SARS-CoV-2 detection

Because repeated lid opening may introduce aerosol contamination of pathogens, several groups have developed approaches to eliminate the risk. For example, Cas12a reagents could be attached to the inner side of the wall (Wang et al., 2019) or the lid (Sun et al., 2021) of test tubes that contain RT-RPA reagents. Upon completion of RT-RPA reaction, Cas12a reagents were introduced into the RT-RPA reaction product by centrifugation. Although these methods appeared feasible to integrate RT-RPA and CRISPR reaction in one test tube, in practice due to the lack of physical containment Cas12a reagents might be introduced into RT-RPA reaction during the interval mixing step, which is required for RT-RPA to maximize amplification efficiency especially with low-copy samples (Lillis et al., 2016). Recently, a one-shot reaction has been developed to integrate isothermal amplification and CRISPR reactions into one step with an approximate detection time of 1 h (Joung et al., 2020).

**Figure 2:**
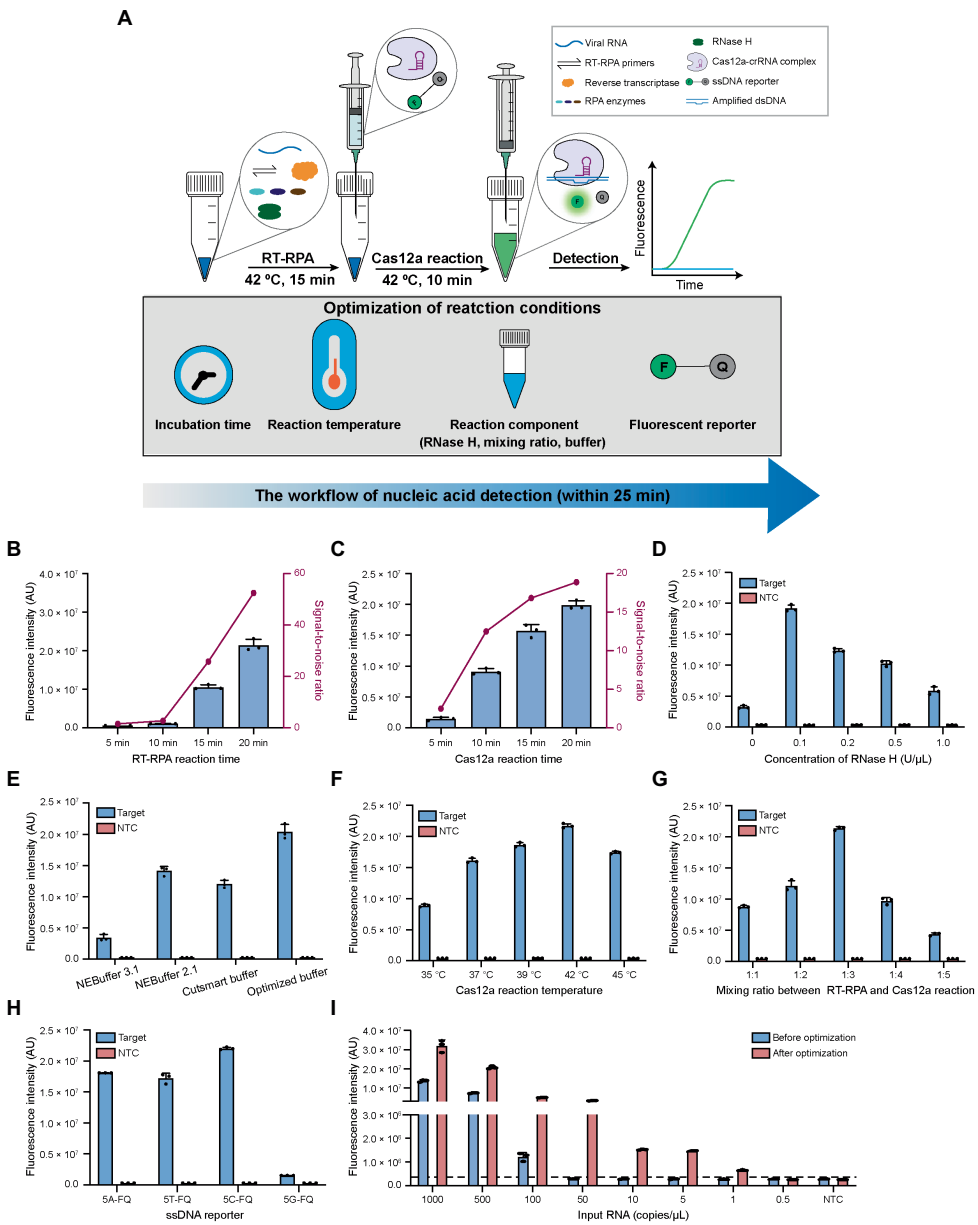
Optimization of the reaction conditions for CALIBURN-v2. **(A)** Schematic presentation of CALIBURN-v2 for closed-lid pathogen detection, showing reaction conditions to be optimized. **(B,C)** The effects of incubation time of RT-RPA **(B)** and Cas12a **(C)** reaction on the final signal readout. **(D)** Screening the optimal concentrations for RNase H. **(E)** Comparison of the assay efficiency using different Cas12a reaction buffers. **(F–H)** Effect of Cas12a reaction temperature **(F)**, the mixing ratio between RT-RPA and Cas12a reaction **(G)**, and the sequences of ssDNA reporters **(H)** on the assay performance. **(I)** Comparison of the sensitivity of the assay before and after optimization. The black dotted line represents the threshold, which is set as the mean of NTC plus 3-fold standard deviation (SD). Target, SARS-CoV-2 IVT N gene RNA (1 nM). NTC, non-template control. The data from three biological replicates are shown as mean ± SD.

In this study, we propose to implement a closed-lid, streamlined RT-RPA and Cas12a reaction system by loading syringes carrying pre-made Cas12a master reaction on to the RT-RPA reaction tubes. This method was optimized upon a previously reported Cas12a-based diagnostic platform for respiratory pathogens, which was referred to as CALIBURN for Cas12a-Linked Beam Unlocking Reaction. In this study, we further optimized CALIBURN for the RT-RPA primers, crRNA and reaction conditions and referred the optimized method as CALIBURN-v2 in the subsequent sections. The goal of the optimization efforts in CALIBURN-v2 was to eliminate the risk of aerosol contamination while retaining high-efficiency detection (Figure 2A).

### Optimization of reaction time of CALIBURN-v2

In the previous study, CALIBURN-v1 contains a 30 min RT-RPA reaction and a 30 min Cas12a fluorescence activation reaction (Wang et al., 2021). Recent study has highlighted the turnaround of COVID-19 diagnosis (Larremore et al., 2021) and several studies have revealed the feasibility of reducing the incubation time for RT-RPA and Cas12a reactions (Qing et al., 2020; Park et al., 2021; Zhang et al., 2021). Herein, we examined the effects of the incubation time of RT-RPA and Cas12a reactions on the fluorescence signal of CALIBURN-v2. It was found that the fluorescence increased with the increase of incubation time of RT-RPA reaction during a period of 20 min. The most notable increase of signal-to-noise ratio occurred between 10 and 15 min (Figure 2B; Supplementary Figure S2A). We therefore selected 15 min as the optimal time for RT-RPA reaction. Similarly, for the Cas12a reaction the greatest increase of signal-to-ratio appeared between 5 and 10 min and thus 10 min was selected as the optimal reaction time (Figure 2C; Supplementary Figure S2B). This optimization could reduce the reaction time from 60 min in CALIBURN-v1 (Wang et al., 2021) to 25 min in CALIBURN-v2 while retaining the overall performance of the detection reaction.

### Determination of the effects of RNase H on CALIBURN-v2

We next sought to determine the rate-limiting step in RT-RPA reaction. By comparing the RPA reaction using RNA or DNA as the input template, we found that the reaction has a 20-fold lower limit-of-detection (LOD) toward input DNA template than toward RNA (5 copies/μL versus 100 copies/μL; Supplementary Figures S3A,B). We thus hypothesized that the reverse transcription process of RNA was the rate-limiting step. We next compared two commonly used reverse transcriptases AMV and ProtoScript II for their efficiencies in CALIBURN-v2. It was found that AMV resulted in stronger fluorescence signal and faster reaction rate than ProtoScript II (Supplementary Figure S3C). The main difference between these two reverse transcriptases was that AMV had RNase H activity, which could specifically degrade RNA in RNA-cDNA hybrid, while ProtoScript II was known to lack the endoribonuclease activity (Ren et al., 2016; Malina et al., 2020). It was possible that the presence of RNase H activity could prompt the hydrolysis of RNA from the RNA-cDNA hybrid duplex, thus facilitating the release of cDNA for subsequent amplification reaction. This hypothesis was further supported by the observation that the presence of additional RNase H in ProtoScript II-enabled RT-RPA could markedly improve the reaction efficiency with much faster rate to reach the plateau (Supplementary Figure S3D). It was found that the optimal concentration for RNase H was 0.1 U/μL (Figure 2D; Supplementary Figure S3E). Collectively, our results suggested that the RNA-cDNA hybrid during reverse transcription process was the rate-limiting step and that supplementation of low concentration of RNase H could facilitate the downstream amplification reaction by releasing more cDNA template.

### Optimization of reaction buffer in CALIBURN-v2

In our previous study, NEBuffer 3.1 was used for Cas12a reaction, which enabled detection of SARS-CoV-2 with 92% sensitivity in 30 min (Wang et al., 2021). In the present study, we optimized the buffering conditions in Cas12a reaction, aiming to improve the overall efficiency of CALIBURN-v2. It was found that switching the buffer to NEBuffer 2.1 could significantly improve Cas12a reaction efficiency particularly at the point of 10 min (Supplementary Figure S4A), suggesting that NEBuffer 2.1 might enhance the reaction rate. Inspired by these results, we further evaluated the effects of the components of the reaction buffer on the efficiency of Cas12a reaction, including the concentrations of NaCl, MgCl_2_, Tris–HCl, dithiothreitol (DTT) and bovine serum albumin (BSA). The optimal buffer conditions were determined to be: 10 mM Tris–HCl, pH 7.9, 20 mM NaCl, 15 mM MgCl_2_, 0.5 mM DTT and 100 μg/ml BSA (Supplementary Figures S4B–F). We then compared the home-optimized reaction buffer with three commercially available buffers including NEBuffer 3.1, NEBuffer 2.1 and Cutsmart buffer. It was found that the above home-optimized buffer surpassed all the commercial buffers (Figure 2E; Supplementary Figure S5A).

### Optimization of reaction temperatures in CALIBURN-v2

Incubation temperatures of RT-RPA and Cas12a reactions were important factors for the overall efficiency. Previous studies suggested that the optimal reaction temperatures for RT-RPA and Cas12a were 42°C and 37°C, respectively, (Huang et al., 2020; Xiong et al., 2020; Park et al., 2021). Interestingly, it was found in this study that the optimal temperature for Cas12a reaction was 42°C under CALIBURN-v2 reaction conditions (Figure 2F; Supplementary Figure S5B).

### Optimization of the mixing ratio of RT-RPA and Cas12a reactions in CALIBURN-v2

To eliminate the uncapping procedure, Cas12a reaction solutions were pre-made in the syringe and directly introduced into the completed RT-RPA reaction. Under this design, the mixing ratio between the two concerted reactions could be critical because the excess polyethylene glycol (PEG) in the RT-RPA reaction might be inhibitory to Cas12a reaction (Lillis et al., 2016; Kellner et al., 2019; Gong et al., 2021). Therefore, the mixing ratio was varied from 1:1 to 1:5 and it was found that the maximum efficiency was achieved at 1:3 ratio (Figure 2G; Supplementary Figure S5C). Thus, in the subsequent studies CALIBURN-v2 was set as 25 μl of RT-RPA reagent in the initial test tube and 75 μl of Cas12a reaction in the syringe.

### Optimization of the fluorescence reporter sequence in CALIBURN-v2

Despite the widespread application of Cas12a-based detection, very few studies reported the study of the effects of ssDNA reporter on the *trans*-cleavage activity of Cas12a. Herein, we investigated the effects of the base compositions of ssDNA reporter on Cas12a and found that the *trans*-cleavage activity was highest for cytosine ssDNA and lowest for guanine ssDNA (Figure 2H; Supplementary Figure S5D). Thus, subsequent studies were performed with 5C-FQ ssDNA reporter.

### Determination of the LODs of CALIBURN-v2

With all the above parameters optimized, we sought to determine the overall additive benefits of the optimization on improving the LOD of CALIBURN-v2. Using SARS-CoV-2 IVT N gene RNA as the substrate, we found that the LOD of CALIBURN-v2 was decreased from 100 copies/μL to 1 copy/μL after optimization, equivalent to a 100-fold increase in sensitivity (Figure 2I; Supplementary Figures S5E,F). Collectively, these results showed that within a total reaction time of 25 min, CALIBURN-v2 could achieve an optimum detection sensitivity.

### Development of IoMT-enabled CALIBURN-v2 with smartphone-controlled data collection and data management in cloud server

**Figure 3:**
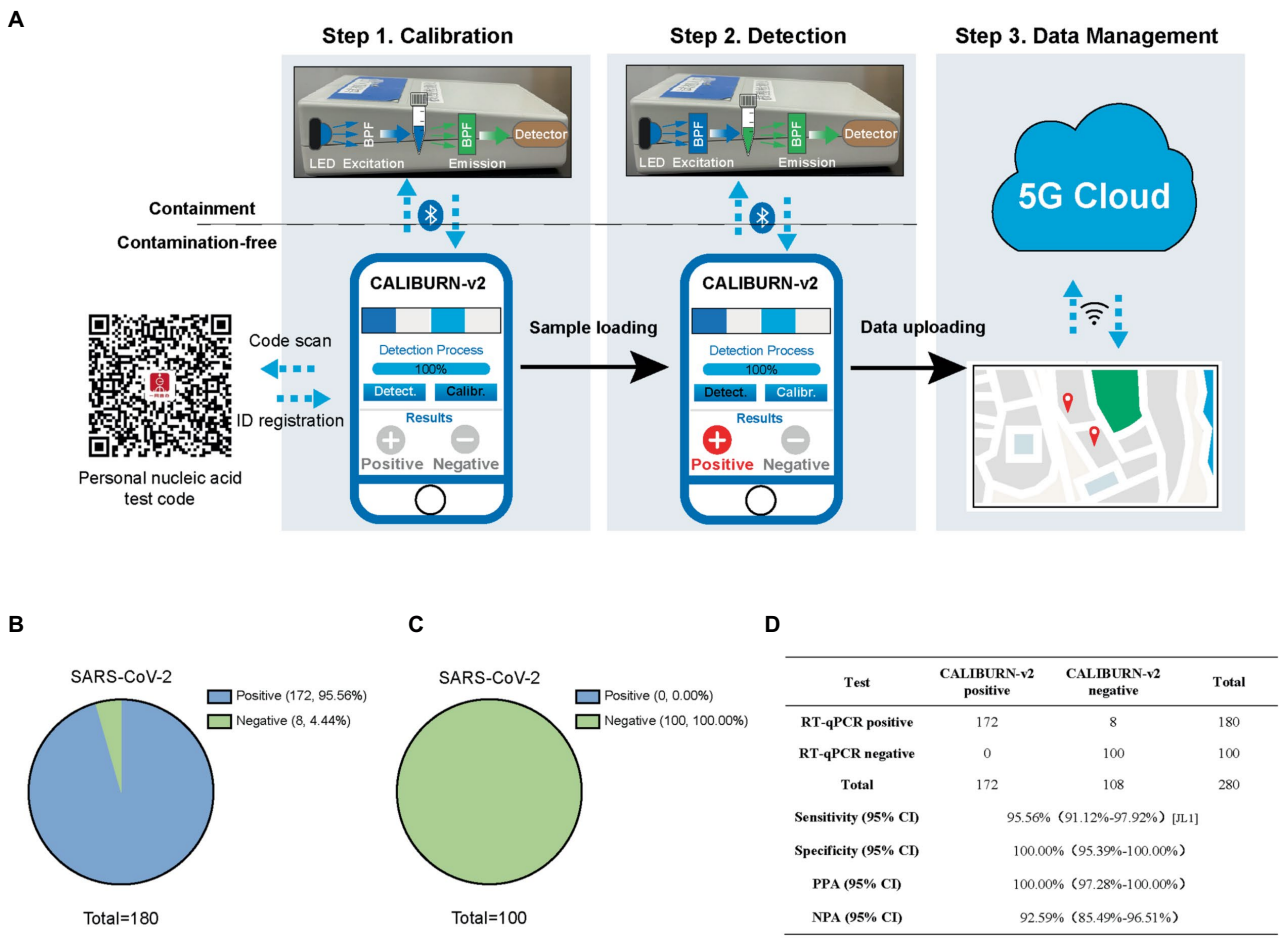
Integration of smartphone-controlled POCT with the closed-lid, CALIBURN-v2. **(A)** Schematic illustration of smartphone-controlled signal readout using the POCT device. The detection results are sent to the 5G cloud for subsequent analysis. The red dots denote the locations of positive cases in the map. **(B)** Sensitivity of CALIBURN-v2 on viral nucleic acid from clinical specimens. **(C)** Specificity of CALIBURN-v2. The RT-RPA primers and crRNAs for SARS-CoV-2 are used to detect nucleic acid samples from healthy donors, respectively. **(D)** Concordance between RT-qPCR and CALIBURN-v2 for SARS-CoV-2 detection. PPA, positive predictive agreement; NPA, negative predictive agreement; CI, confidence interval.

The output of CRISPR-based diagnostic tests could be colorimetry or fluorescence-based signal readout (Ding et al., 2020; Gong et al., 2021; Kaminski et al., 2021; Ooi et al., 2021). Herein, to realize full quantification of the signal from CALIBURN-v2, we developed a smartphone-controlled POCT device (Figure 3A), which could detect the fluorescence in CALIBURN-v2 and convert the fluorescence signal to electrical signal. The process of POCT manipulation contains three consecutive steps. The first step is calibration, where the signal from a control sample without nucleic acid input was collected. The second step is sample information collection, where positive and negative samples were determined by comparing their signals with those of control sample. In the third step, the information is uploaded to a 5G cloud database and synchronized with a mapping server to display the location of the sample determination. The access to the location information is strictly regulated with the consideration of privacy issues. The communication between the device and smartphone is controlled by a Bluetooth-supported application with user interface on the smartphone. The design of smartphone-controlled data collection and management enabled integration of POCT and Internet of Medical Things (IoMT) features for telehealth service and big data analysis (Ding et al., 2019).

Next, we evaluated the performance of IoMT-enabled CALIBURN-v2 using SARS-CoV-2 clinical samples. The results showed that smartphone-controlled CALIBURN-v2 exhibited 95.56% (172 out of 180) sensitivity (Figure 3B) and 100% specificity (100 out of 100; Figure 3C) for SARS-CoV-2. These results correspond to 100% positive predictive agreement (PPA) and 92.59% negative predictive agreement (NPA; Figure 3D).

### Performance of IoMT-enabled CALIBURN-v2 on SARS-CoV-2 variants

**Figure 4:**
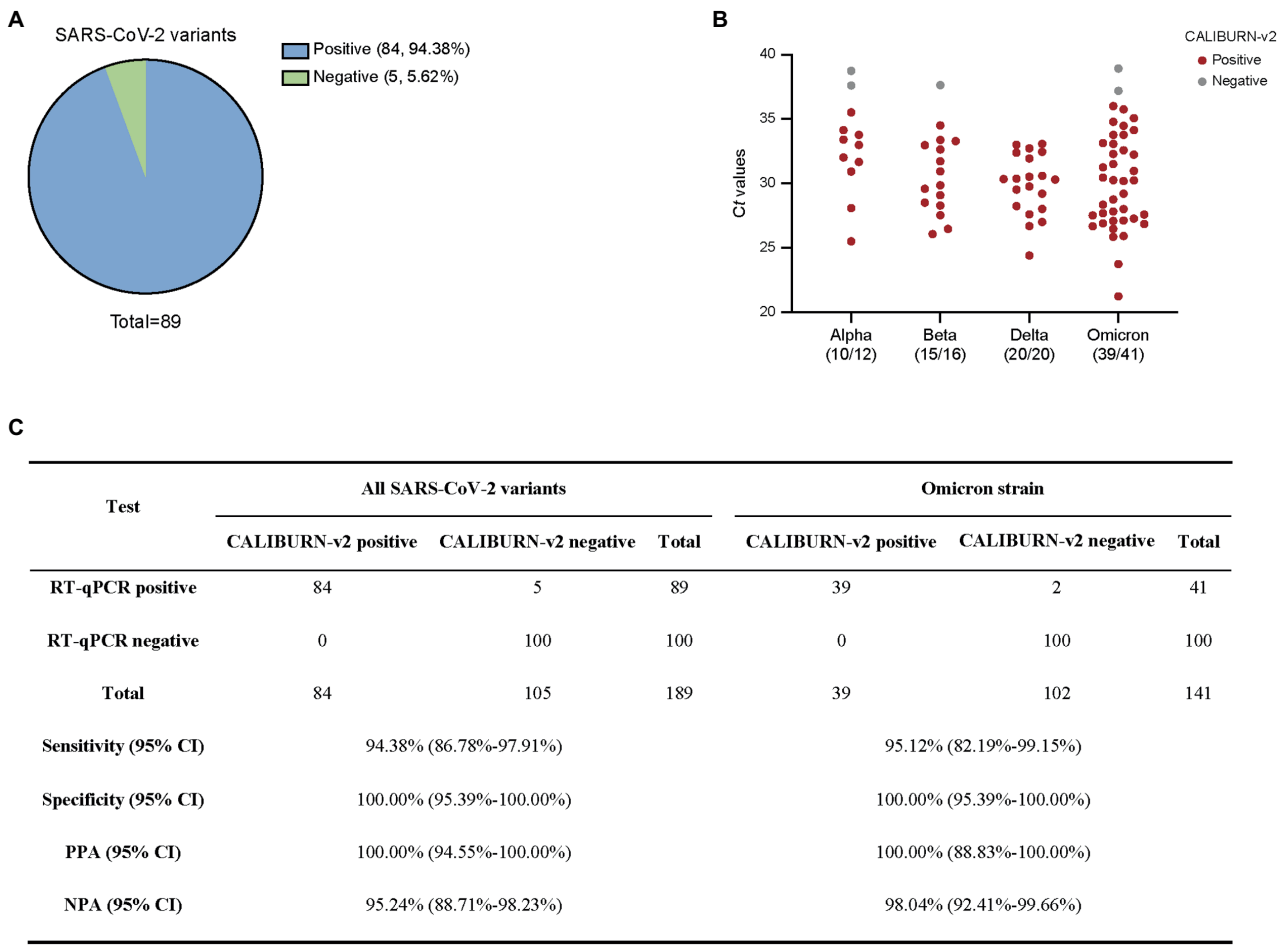
Detection of SARS-CoV-2 variants using smartphone-controlled CALIBURN-v2. **(A)** Overall sensitivity of CALIBURN-v2 on clinical nucleic acid specimens of SARS-CoV-2 variants. **(B)** Sensitivity of CALIBURN-v2 on different SARS-CoV-2 variants and the correlations with RT-qPCR C*t* values. The ratios of positive samples with each variants are indicated. C*t* values are obtained from the RT-qPCR assay for SARS-CoV-2 N gene. NTC, non-template control. **(C)** Concordance between RT-qPCR and CALIBURN-v2 for SARS-CoV-2 variant detection. PPA, positive predictive agreement; NPA, negative predictive agreement; CI, confidence interval.

In the current study, we collected Alpha, Beta, Delta and Omicron SARS-CoV-2 clinical samples and determined the efficiency of IoMT-enabled CALIBURN-v2 on these variants. Similar to the results with SARS-CoV-2 wild type, CALIBURN-v2 exhibited 94.38% overall sensitivity toward all variants (84 out of 89 samples; Figure 4A) and 95.12% sensitivity toward Omicron variant (39 out of 41 samples; Figure 4B). The PPA and NPA values toward all variants or Omicron are similar to those with SARS-CoV-2 wild type (Figure 4C). It has been noted that the negative samples that CALIBURN-v2 failed to detect appeared to all have C*t* values of higher than 37 (Figure 4B). It has been found that patients with C*t* values greater than 34 were not contagious and could be allowed for quarantine termination (La Scola et al., 2020).

### Performance of IoMT-enabling CALIBURN-v2 on influenza viruses

To demonstrate the general applicability of CALIBURN-v2, we designed RT-RPA primers and crRNAs for influenza viruses. Degenerate nucleotides were used in RT-RPA primers to ensure the coverage of different influenza viruses. We analyzed the genomic sequences of influenza A viruses (IAVs) and influenza B viruses (IBVs) and identified conserved regions in the M gene of IAV and HA gene of IBV, respectively, (Supplementary Figures S6A,B; Supplementary Table S1).

We designed one pair of primers each for IAV and IBV and screened four crRNAs. It was found that IAV-M-crRNA1 and IBV-HA-crRNA3 exhibited the highest fluorescence signal and reaction rate, with minimum time for reaching the reaction plateau (Supplementary Figure S7A). The results showed that IAV primers and crRNA could specifically detect common human IAVs including H1N1, H2N3 and H9N2, with little cross reactivity with coronaviruses or IBVs (Supplementary Figure S7B). Likewise, IBV primers and crRNA could detect the Yamagata and Victoria lineages of IBV with high specificity (Supplementary Figure S7B). Collectively, our results showed that optimized design of RT-RPA primers and Cas12a crRNA could enable specific detection of IAVs and IBVs, which was necessary for instructing subsequent clinical treatment particularly considering the occurrence of co-infection of SARS-CoV-2 and influenza viruses (Ozaras et al., 2020).

**Figure 5:**
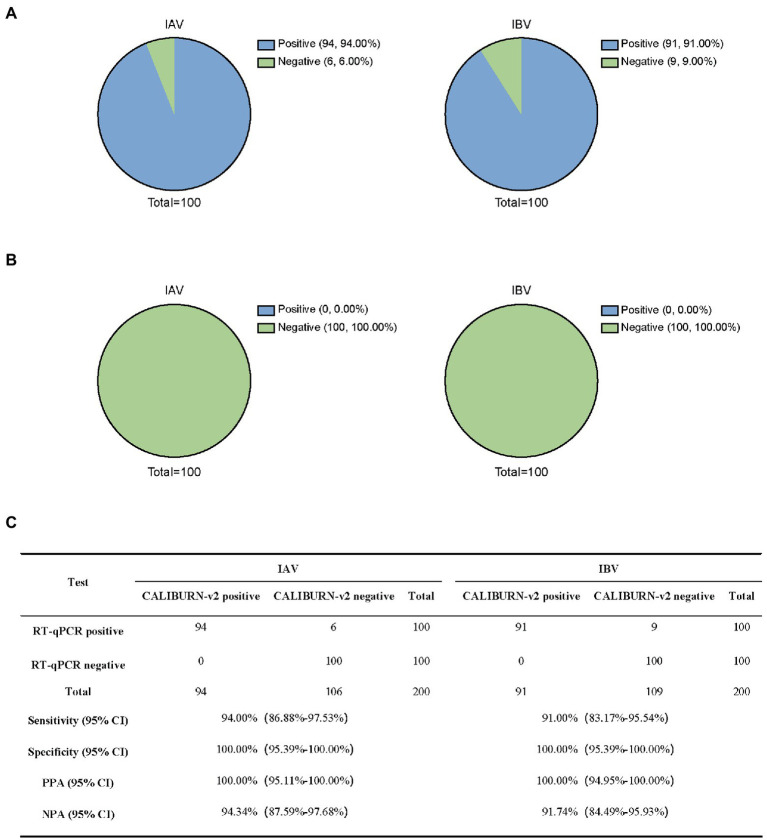
Detection of influenza virus using CALIBURN-v2. **(A,B)** Sensitivity **(A)** and specificity **(B)** of CALIBURN-v2 for influenza virus detection. **(C)** Concordance between RT-qPCR and CALIBURN-v2 for IAV and IBV. The RT-RPA primers and crRNAs for IAV and IBV are used to detect nucleic acid samples from healthy donors, respectively. PPA, positive predictive agreement; NPA, negative predictive agreement; CI, confidence interval.

Next, we analyzed RT-qPCR-confirmed IAV samples (100) and IBV samples (100). CALIBURN-v2 exhibited 94.00 and 91.00% sensitivity to IAV and IBV, respectively, (Figure 5A) and 100% specificity for both pathogens (Figure 5B). These results corresponded to 100% PPA for both pathogens and NPA values of 94.34% for IAV and 91.74% for IBV, respectively (Figure 5C).

## Discussion

In this study, we established a rapid and highly efficient CRISPR diagnostics for the detection of SARS-CoV-2 VOCs in a closed-lid mode. This platform, referred to as CALIBURN-v2, has been extensively optimized to improve the detection efficiency, reaction time and LODs over previous platforms.

Although some previous studies reported closed-lid approaches for CRISPR diagnostics, these studies typically avoided repeated capping and uncapping procedures by attaching Cas12a reaction components to the inner side of tube lid or tube wall and mixing the RT-RPA and Cas12a reactions using centrifugation. In practice, these methods are liable to external perturbation due to the lack of physical separation. In our study, we adopted a needle-penetrating approach which could maximize the consistency of detection performance.

To improve the performance of CALIBURN-v2 in closed-lid mode, we optimized a series of reaction parameters including those that have not been previously explored, such as the concentration of RNase H. Despite small improvements on each single parameter, the final reactions with all combined optimized parameters in fact improved the detection efficiency in closed-lid mode by 100 folds. This remarkable improvement allowed for highly efficient detection of all SARS-CoV-2 VOCs. These improvements were also necessary for providing compability with downstream portable device containing IoMT function. Moreover, further efforts can be made to develop device and IoMT system can be used to collect the lateral flow signals, likely by colorimetric quantification, in addition to the fluorescence signal.

One notable improvement of CALIBURN-v2 over existing CRISPR diagnostics is that the concept of IoMT is introduced. Several earlier studies have reported cell phone-controlled CRISPR detection reaction. However, none of these studies contained cloud server-based data collection and management systems that could facilitate real-time monitoring of the dynamics of COVID-19 pandemic. The fast turnover of data collection and analysis in population scale is important for timely decision-making. Although we focused on the detection of SARS-CoV-2 N gene in this study, previous studies have also shown that CRISPR diagnostics could detect SARS-CoV-2 mutations in regions other than N gene. Similar to the results shown in this study, CRISPR detection of S gene could also support specific diagnosis of SARS-CoV-2 over other coronaviruses (Ma et al., 2020). Most importantly, we have shown that the IoMT-enabled CALIBURN-v2 exhibits around 95% sensitivity and 100% specificity when detecting different pathogens. In future studies, the performance of this system could be further evaluated in expanded categories of pathogens.

In addition, although most nucleic acid testing kits for SARS-CoV-2 have two target genes, existing marketed CRISPR diagnostic testing kits have only one target gene. One possible reason for the authority to allow one target gene is that CRISPR diagnostics typically require a coupled RPA reaction, which provides another dimension of specificity from primer binding in addition to crRNA targeting in the CRISPR reaction. Nevertheless, strategies have been developed for CRISPR diagnostics to enable multiplexable detection (Jia et al., 2021). One of the future directions to improve CALIBURN is to integrate multiple gene targets for increased specificity. Furthermore, like most nucleic acid diagnostic testing methods the performance of CRISPR diagnosis depends on the efficiency of nucleic acid extraction. Endeavors should be made to develop and optimize CRISPR-compatible extraction methods.

Finally, our study has provided a feasible approach to IoMT-enabled CRISPR diagnostics in a closed-lid reaction mode and has demonstrated optimal performance with clinical samples that is comparable to markted products. We envision that our study can prompt the application of the versatile CRISPR diagnostics technology.

## Data availability statement

The original contributions presented in the study are included in the article/Supplementary material, further inquiries can be directed to the corresponding authors.

## Ethics statement

All clinical samples were collected without identifiable personal information and used solely for molecular diagnosis purpose. The study was approved by the Ethics Committees of Ruijin Hospital and ShanghaiTech University.

## Author contributions

HL, WZ, SL, JQ, and JL: conceptualization. HL, WZ, SL, YW, DW, and LX: methodology. HL and WZ: investigation. HL and JL: writing–original draft. HL, ZT, SW, JQ, and JL: writing–reviewing and editing. WZ, SW, JQ, and JL: funding acquisition. SL and DW: resources. ZT, SW, JQ, and JL: supervision. All authors contributed to the article and approved the submitted version.

## Funding

This work was financially supported by Zhangjiang National Innovation Demonstration Zone (ZJ2020-ZD-004 to JL and JQ), Zhejiang University special scientific research fund for COVID-19 prevention and control (2020XGZX011 to JQ), National Key Research and Development Program of China (2018YFE0102400 to JQ), Shanghai Frontiers Science Center for Biomacromolecules and Precision Medicine at ShanghaiTech University (to JL), Science and Technology Commission of Shanghai Municipality (19JC1413000 and 19430750600 to JQ), Cultivation Project of Shanghai Major Infectious Disease Research Base (20dz2210500 to JQ), Key Laboratory of Emergency Prevention, Diagnosis and Treatment of Respiratory Infectious Diseases, Shanghai (20dz2261100 to JQ), Medicine and Engineering Interdisciplinary Research Fund of Shanghai Jiao Tong University (19×190020171 to JQ), National Innovative Research Team of High-level Local Universities in Shanghai, and ShanghaiTech University Startup Fund (2019F0301-000-01 to JL).

## Conflict of interest

The authors declare that the research was conducted in the absence of any commercial or financial relationships that could be construed as a potential conflict of interest.

## Publisher’s note

All claims expressed in this article are solely those of the authors and do not necessarily represent those of their affiliated organizations, or those of the publisher, the editors and the reviewers. Any product that may be evaluated in this article, or claim that may be made by its manufacturer, is not guaranteed or endorsed by the publisher.
