# Supplementary material for: Internet of medical things-enabled CRISPR diagnostics for rapid detection of SARS-CoV-2 variants of concern
Source: Front Microbiol. 2022 Nov 18;13:1070940. doi: 10.3389/fmicb.2022.1070940 (PMC9715597; doi:10.3389/fmicb.2022.1070940)
Supplement: Supplementary file 1 [file Data_Sheet_1.docx]

***Supplementary Materials***

**Supplementary Figures**

**
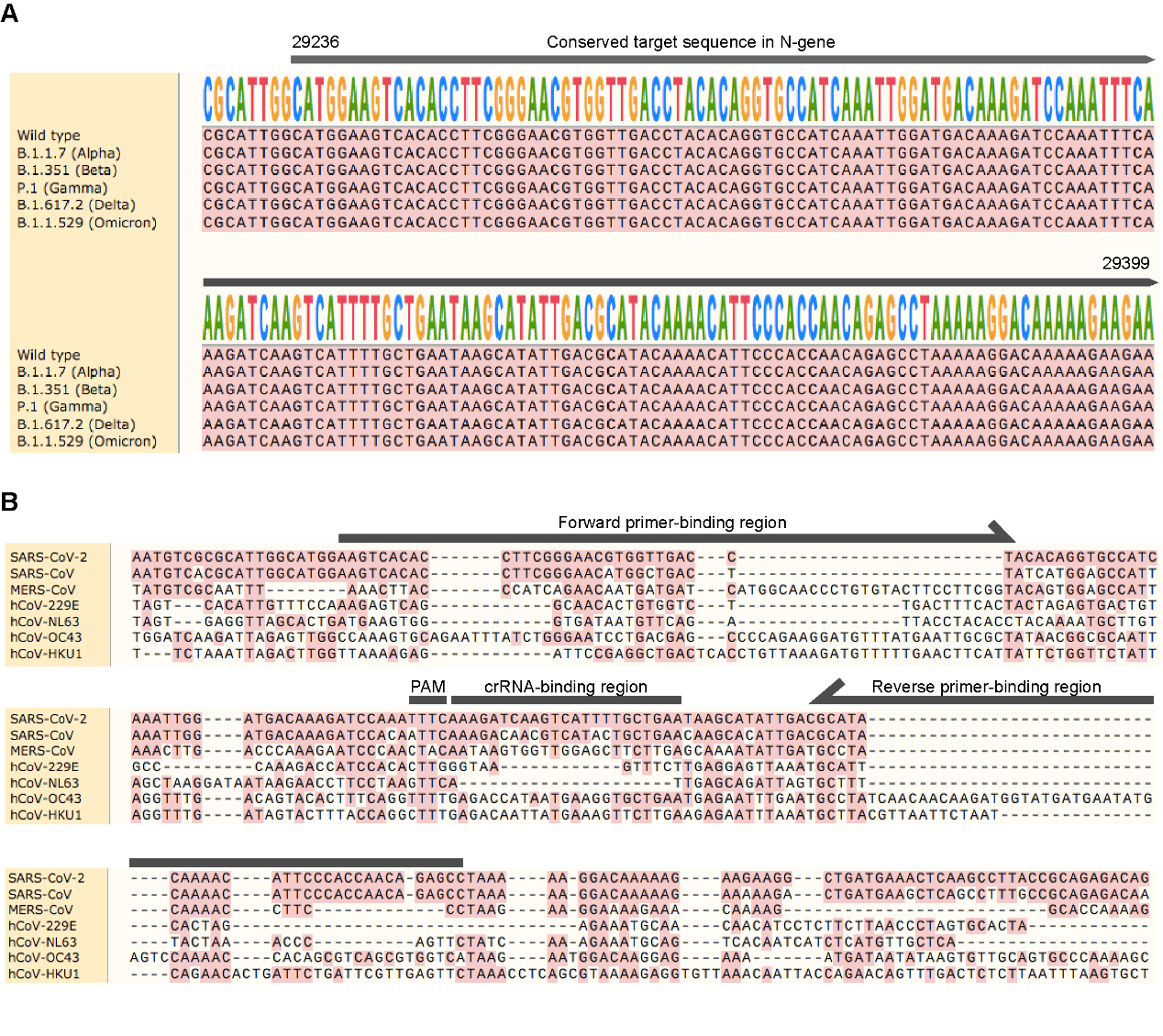
**

**Figure S1. Design of RT-RPA primers and crRNAs. (A**) Alignment of the N gene of SARS-CoV-2 wild type and VOCs (Alpha, Beta, Gamma, Delta and Omicron). Gray arrow denotes the conserved target sequence. (**B)** Alignment of SARS-CoV-2 with other human coronaviruses including SARS-CoV, MERS-CoV, hCoV-229E, hCoV-NL63 hCoV-OC43 and hCOV-HKU1, showing the primer- and crRNA-binding regions in SARS-CoV-2.


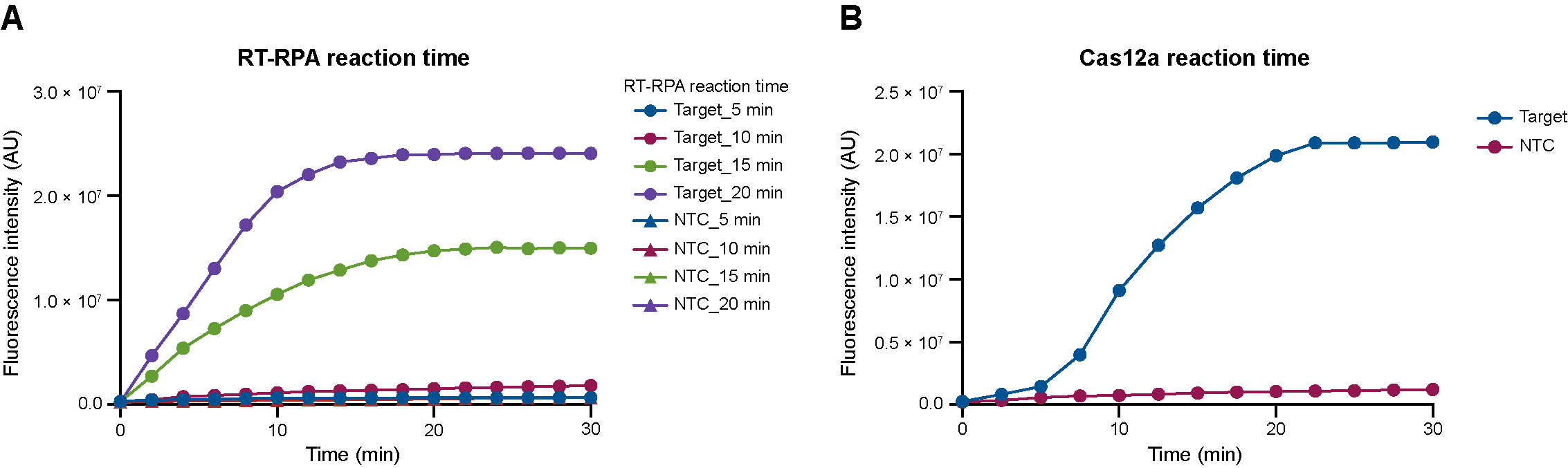


**Figure S2.** **Determination of optimal incubation time for streamlined RT-RPA and Cas12a reactions.** **(A)** Real-time fluorescence kinetics of Cas12a reaction for determination of optimal RT-RPA reaction time. **(B)** Real-time fluorescence kinetics of Cas12a reaction for determination of optimal Cas12a reaction time. Target, SARS-CoV-2 IVT N gene RNA (1 nM). NTC, non-template control.


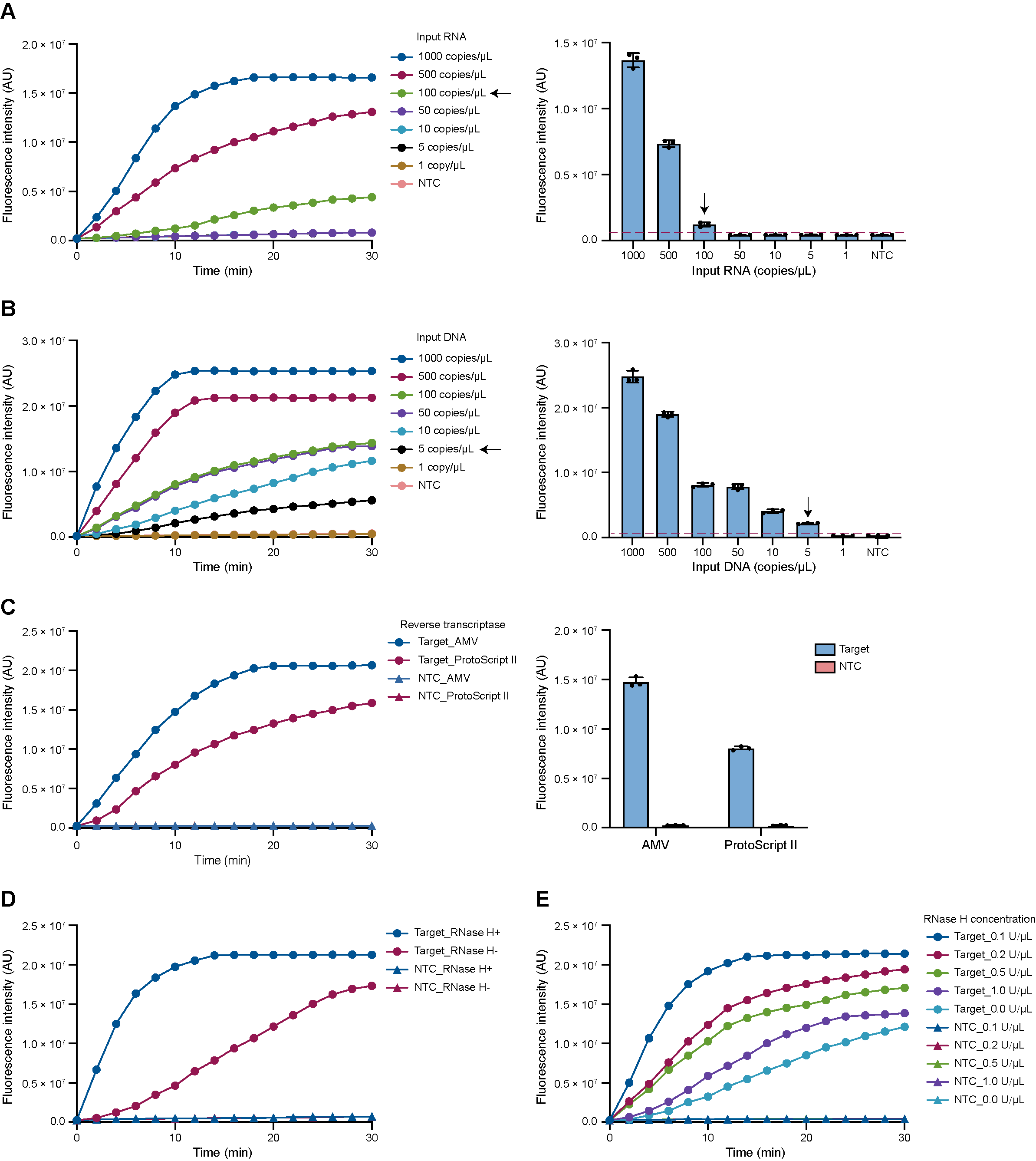


**Figure S3. The effects of RNase H on RT-RPA performance.** **(A, B)** Determination of LOD for Cas12a reaction combined with RT-RPA using purified SARS-CoV-2 IVT N gene RNA **(A)** or SARS-CoV-2 N gene RT-PCR products (DNA) **(B)** as input target. Arrows denote LODs, the red dotted line represents the threshold, which is set as the mean of NTC plus 3-fold standard deviation (SD). **(C)** Comparison of the performance of AMV and ProtoScript II reverse transcriptase on the detection of SARS-CoV-2 IVT N gene RNA. For **(A-C),** the real-time (left panel) and 10-min point (right panel) fluorescence signals are shown. **(D)** Real-time fluorescent kinetics of Cas12a reaction using ProtoScript II reverse transcriptase in the presence and absence of additional RNase H in RT-RPA. **(E)** Real-time fluorescent kinetics of Cas12a reaction with various concentrations of RNase H in ProtoScript II-enabled RT-RPA. For **(C-E),** target refers to SARS-CoV-2 IVT N gene RNA (1 nM). NTC, non-template control. The data from three biological replicates are shown as mean ± SD.


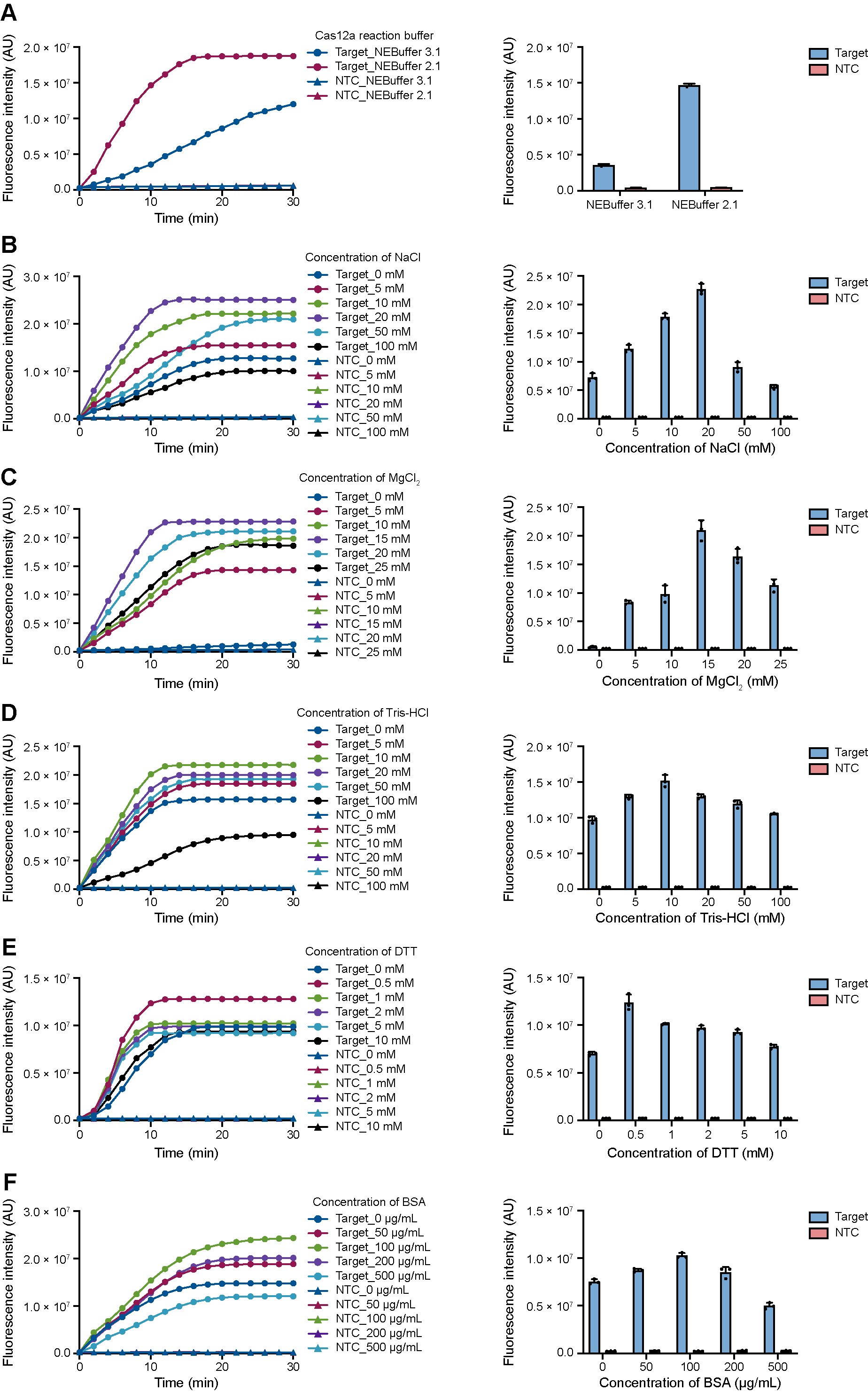


**Figure S4. Optimization of reaction buffer for Cas12a reaction. (A)** Comparison of NEBuffer 3.1 and 2.1 for their efficiencies in Cas12a reaction. **(B-F)** Evaluation of the Cas12a reaction efficiency under the various concentrations of NaCl **(B)**, MgCl_2_ **(C)**, Tris-HCl **(D)**, DTT **(E)** and BSA **(F)**. The real-time (left panel) and 10-min point (right panel) fluorescence signals are shown. Target, SARS-CoV-2 IVT N gene RNA (1 nM). NTC, non-template control. The data from three biological replicates are shown as mean ± standard deviation (SD).


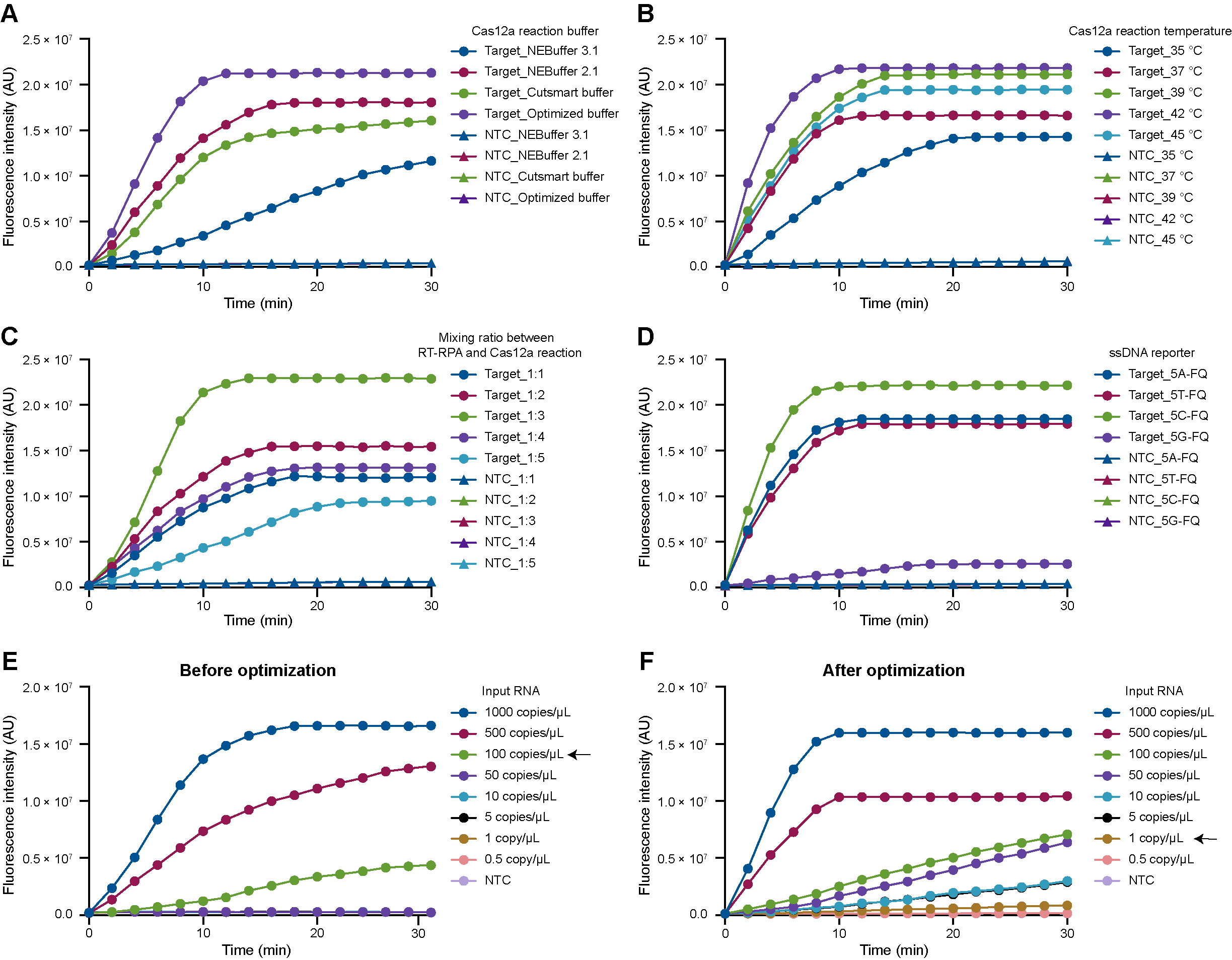


**Figure S5. Optimization of reaction conditions for CALIBURN-v2. (A-D)** Real-time fluorescence kinetics of Cas12a reaction with different reaction buffers **(A)**, reaction temperatures **(B)**, mixing ratios between RT-RPA and Cas12a reaction **(C)** and sequences of ssDNA reporters **(D)**. **(E, F)** The sensitivity of SARS-CoV-2 detection before **(E)** and after optimization **(F)**. Arrows denote LODs, as defined by the mean of NTC plus 3-fold standard deviation. Target, SARS-CoV-2 IVT N gene RNA (1 nM). NTC, non-template control.


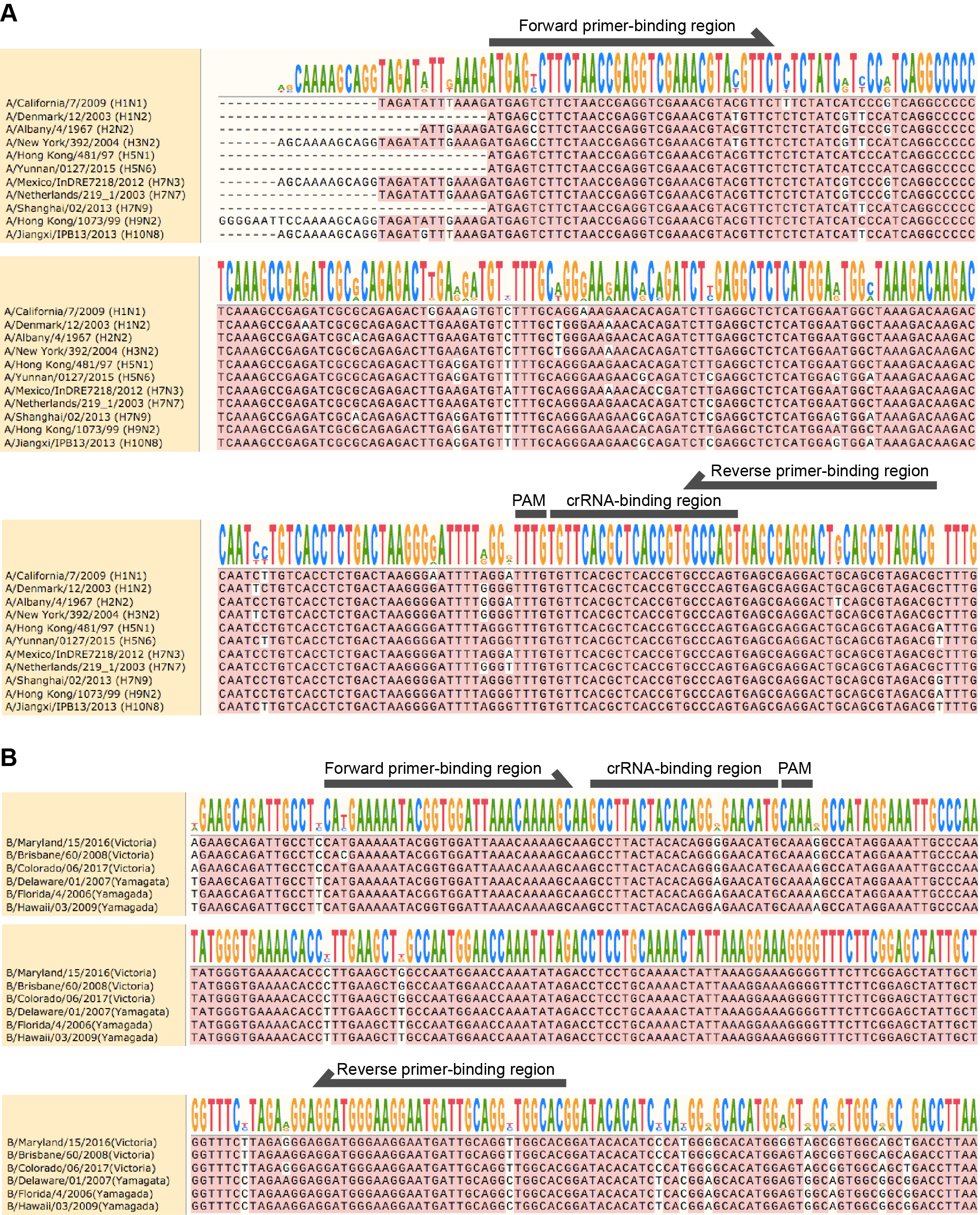


**Figure S6.** Target sequences for influenza virus detection. (**A, B)** Alignment of primer- and crRNA-binding regions of IAVs **(A)** and IBVs **(B)**.


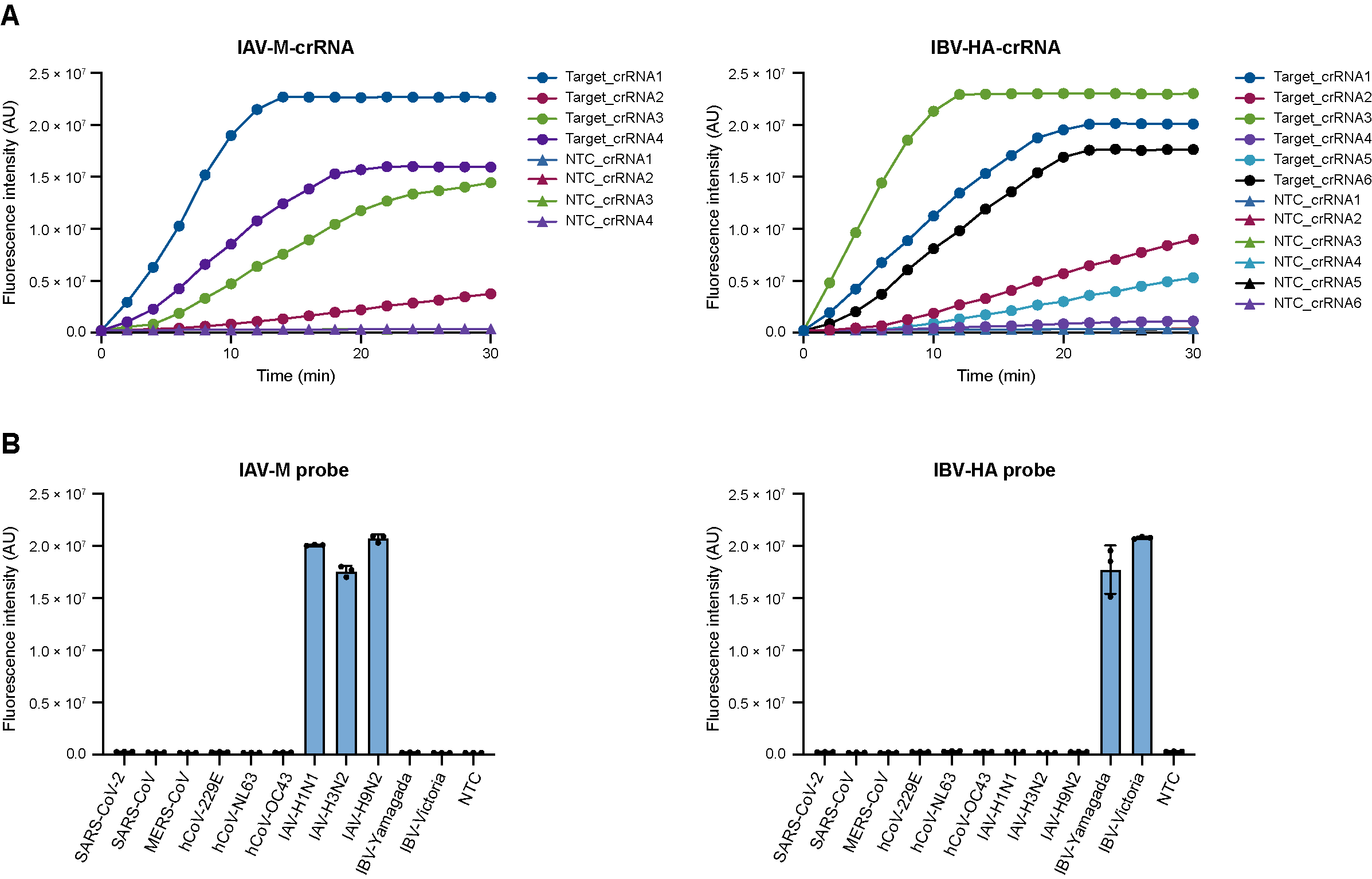


**Figure S7.** **Specificity of influenza virus detection.** (**A)** Kinetics of the Cas12a fluorescence reaction for screening IAV and IBV crRNAs. **(B)** Fluorescence signal is collected after 10 min of Cas12a reaction, with the input viral copies in the range of 10^6^-10^7^ per reaction. The RT-RPA primers are IAV-M-RPA-F/IAV-M-RPA-R for IAV, and IBV-HA-RPA-F/IBV-HA-RPA-R for IBV, respectively. The crRNAs are IAV-M-crRNA1 for IAV, and IBV-HA-crRNA3 for IBV, respectively. NTC, non-template control. The data from three biological replicates are shown as mean ± standard deviation (SD).

**Supplementary Tables**

**Table S1. Primers, probes and crRNAs used in this study**

| **Name** | **Sequence (5' to 3')** |
| --- | --- |
| SARSCoV2-N-RPA-F1 | CATGGAAGTCACACCTTCGGGAACGTGGTT |
| SARSCoV2-N-RPA-F2 | ATGGAAGTCACACCTTCGGGAACGTGGTTG |
| SARSCoV2-N-RPA-F3 | AAGTCACACCTTCGGGAACGTGGTTGACCT |
| SARSCoV2-N-RPA-F4 | GAAGTCACACCTTCGGGAACGTGGTTGACCTA |
| SARSCoV2-N-RPA-R1 | CCTTCTTCTTTTTGTCCTTTTTAGGCTCTG |
| SARSCoV2-N-RPA-R2 | GGCTCTGTTGGTGGGAATGTTTTGTATGCG |
| SARSCoV2-N-RPA-R3 | TCTTCTTTTTGTCCTTTTTAGGCTCTGTTGG |
| SARSCoV2-N-RPA-R4 | CTTCTTTTTGTCCTTTTTAGGCTCTGTTGGTG |
| IAV-M-RPA-F | ATGAGYCTTCTAACYGARGTCGAAACGTACGTTC |
| IAV-M-RPA-R | CGTCTACGCTGCAGTCCYCGCTCACTGGGC |
| IBV-HA-RPA-F | CAYGAAAAATACGGTGGATTAAACAAAAGC |
| IBV-HA-RPA-R | CGTGCCARCCTGCAATCATTCCTTCCCATC |
| SARSCoV2-N-RTqPCR-F | CCCTGTGGGTTTTACACTTAA |
| SARSCoV2-N-RTqPCR-R | ACGATTGTGCATCAGCTGA |
| SARSCoV2-N-RTqPCR-P | /FAM/CCGTCTGCGGTATGTGGAAAGGTTATGG/BHQ1/ |
| IAV-M-RTqPCR-F | GACCRATCCTGTCACCTCTGAC |
| IAV-M-RTqPCR-R | GGGCATTYTGGACAAAKCGTCTACG |
| IAV-M-RTqPCR-P | /FAM/TGCAGTCCTCGCTCACTGGGCACG/BHQ1/ |
| IBV-HA-RTqPCR-F | TCCTCAACTCACTCTTCGAGCG |
| IBV-HA-RTqPCR-R | CGGTGCTCTTGACCAAATTGG |
| IBV-HA-RTqPCR-P | /FAM/CCAATTCGAGCAGCTGAAACTGCGGTG/BHQ1/ |
| T7-3G primer | GAAATTAATACGACTCACTATAGGG |
| SARSCoV2-N-crRNA1 | UAAUUUCUACUAAGUGUAGAUAUGGCACCUGUGUAGGUCAACCA |
| SARSCoV2-N-crRNA2 | UAAUUUCUACUAAGUGUAGAUUCAUCCAAUUUGAUGGCACCUGU |
| SARSCoV2-N-crRNA3 | UAAUUUCUACUAAGUGUAGAUAAAGAUCAAGUCAUUUUGCUGAA |
| SARSCoV2-N-crRNA4 | UAAUUUCUACUAAGUGUAGAUUAUGCGUCAAUAUGCUUAUUCAG |
| IAV-M-crRNA1 | UAAUUUCUACUAAGUGUAGAUUGUUCACGCUCACCGUGCCCAGU |
| IAV-M-crRNA2 | UAAUUUCUACUAAGUGUAGAUAGGGGGCCUGACGGGACAAUAGA |
| IAV-M-crRNA3 | UAAUUUCUACUAAGUGUAGAUCAGGGAAAAACACCGAUCUUGAG |
| IAV-M-crRNA4 | UAAUUUCUACUAAGUGUAGAUGCCAUUCCAUGAGCGCCUCAAGA |
| IBV-HA-crRNA1 | UAAUUUCUACUAAGUGUAGAUCAUGUUCCCCUGTGUAGUAAGGC |
| IBV-HA-crRNA2 | UAAUUUCUACUAAGUGUAGAUCUAUGGCCUUUGCAUGUUCCCCU |
| IBV-HA-crRNA3 | UAAUUUCUACUAAGUGUAGAUCAUGUUCUCCUGUGUAGUAAGGC |
| IBV-HA-crRNA4 | UAAUUUCUACUAAGUGUAGAUGUUCCAUUGGCCAGCUUCAAGGG |
| IBV-HA-crRNA5 | UAAUUUCUACUAAGUGUAGAUAUAGUUUUGCAGGGGGUCUAUAU |
| IBV-HA-crRNA6 | UAAUUUCUACUAAGUGUAGAUCUUUAAUAGUUUUGCAGGGGGUC |
| 5A-FQ-reporter | /FAM/AAAAA/BHQ1/ |
| 5T-FQ-reporter | /FAM/TTTTT/BHQ1/ |
| 5C-FQ-reporter | /FAM/CCCCC/BHQ1/ |
| 5G-FQ-reporter | /FAM/GGGGG/BHQ1/ |

**Note:** Degenerate bases are Y for C or T, R for A or G and K for G or T.
